# Extended Spectrum Beta-lactamase Detection in Gram-negative Bacilli of Nosocomial Origin

**DOI:** 10.4103/0974-777X.56247

**Published:** 2009

**Authors:** Dechen C Tsering, Shyamasree Das, Luna Adhiakari, Ranabir Pal, Takhellambam SK Singh

**Affiliations:** *Department of Microbiology, Sikkim-Manipal Institute of Medical Sciences(SMIMS) and Central Referral Hospital (CRH), Gangtok, Sikkim, India*; 1 *Department of Community Medicine, Sikkim-Manipal Institute of Medical Sciences(SMIMS) and Central Referral Hospital (CRH), Gangtok, Sikkim, India*

**Keywords:** ESBL, Gram negative bacilli, Nosocomial

## Abstract

**Background:**

Resistance to third generation cephalosporins by acquisition and expression of extended spectrum beta lactamase (ESBL) enzymes among gram-negative bacilli is on a rise. The presence of ESBL producing organisms significantly affects the course and outcome of an infection and poses a challenge to infection management worldwide.

**Materials and Methods:**

In the period from June 2007 to 2008, we collected 1489 samples from patients suspected of nosocomial infection. The isolates were identified based on colony morphology and biochemical reaction. Gram negative bacilli resistant to third generation cephalosporins were tested for ESBL by double disc synergy test (DDST- a screening test)and then phenotypic confirmatory test. Antimicrobial susceptibility testing was done by modified Kirby Bauer disc diffusion method.

**Results:**

From the sample of 238 gram-negative bacilli, we isolated *Escherichia coli, Pseudomonas aeruginosa, Klebsiella pneumoniae, Citrobacter freundii, Proteus mirabilis, Morganella morganii* and *Enterobacter cloacae*. Following both methods, 34% isolates were ESBL-positive. The ESBL producing isolates were significantly resistant (p < 0.01) to ampicillin, piperacillin, piperacillin/tazobactam, trimethoprim/sulfamethoxazole, tetracycline, ciprofloxacin and gentamicin as compared to non-ESBL producers. Multidrug resistance was significantly (p < 0.01) higher (69.14%) in ESBL positive isolates than non-ESBL isolates (21.66%).

**Conclusion:**

High prevalence of ESBL in our hospital cannot be ignored. ESBL producers can be detected by DDST and phenotypic confirmatory test with equal efficacy. The sensitivity of screening test improved with the use of more than one antibiotic and addition of one or two antibiotics would not increase cost and labor. We recommend DDST using multiple antibiotics in all microbiology units as a routine screening test.

## INTRODUCTION

Since the first description of plasmid-mediated extended spectrum beta lactamase (ESBL) in 1983, ESBL-producing gram-negative organisms have posed a significant threat to hospitalized patients due to their hydrolyzing activity against extended spectrum cephalosporins often employed in the treatment of hospital-acquired infections. Detection of organisms harboring ESBLs provides clinicians with helpful information. Treatment of infections caused by ESBL-producing organisms with extended-spectrum cephalosporins or aztreonam may result in treatment failure even when the causative organisms appear to be susceptible to these antimicrobial agents by routine susceptibility testing.[1,2] In addition, patients colonized or infected with ESBL-producing organisms should be placed under contact precautions to avoid hospital transmission.[3] These benefits warrant the detection of ESBL-producing organisms in clinical laboratories. They can be found in a variety of *Enterobacteriaceae* species; however, majority of the ESBL producing strains are *Klebsiella pneumoniae, Klebsiella oxytoca* and *Escherichia coli*. They have also been found in *Pseudomonas aeruginosa* and other *Enterobacteriaceae* strains like *Enterobacter, Citrobacter, Proteus, Morganella morganii, Serratia marsescens, Burkholderia cepacia and Capnocytophaga ochracea*.[4,5]

Several phenotypic tests for detection of ESBL-producing organisms have been developed since the 1980s. All methods utilize the two characteristics of ESBLs: reduction of susceptibility to extended-spectrum cephalosporins and inhibition by clavulanate. The CLSI (Clinical and Laboratory Standards Institute) recommends screening of *E. coli*, *K. pneumoniae,* and *K. oxytoca* (and *Proteus mirabilis*, if clinically relevant such as bacteremic isolates) for potential production of ESBL. The CLSI method for ESBL detection consists of the initial screen test and phenotypic confirmatory test.[6] Susceptibilities to more than one of cefpodoxime, ceftazidime, ceftriaxone, cefotaxime, and aztreonam are evaluated using disk diffusion or broth dilution method in the initial screen test. A decrease in susceptibilities to one or more antibiotics tested may indicate production of ESBLs and warrant performance of the subsequent phenotypic confirmatory tests.

In phenotypic confirmatory tests, susceptibilities to cefotaxime and ceftazidime alone and those with clavulanate are compared using disk diffusion or broth dilution method. If the susceptibility of either antibiotic tested increases significantly (a more than or equal to five mm increase in a zone diameter or a more than or equal to three two-fold decrease in an MIC) in the presence of clavulanate, the result is interpreted as confirmatory of ESBL production.[7]

There is considerable geographical difference in ESBLs in European countries. Within countries, hospital-to-hospital variability in occurrence may also be marked.[8] A large study from more than 100 European intensive care units (ICU) found that the prevalence of ESBLs in Klebsiellae ranged from as low as 3% in Sweden to as high as 34% in Portugal.[9] In Turkey, a survey of *Klebsiella* spp. from ICUs from eight hospitals showed that 58% of 193 isolates harbored ESBLs.[10] Moland and colleagues have shown that ESBL-producing isolates were found in 75% of 24 medical centers in the United States.[11] ESBLs have also been documented in Israel, Saudi Arabia, and a variety of North African countries.[12–14] From China, the figures of ESBL producers vary between 25-40%.[15] National surveys have indicated the presence of ESBLs in 5-8% of E. coli isolates from Japan, Korea, Malaysia and Singapore but 12-24% of isolates from Thailand, Taiwan, Philippines and Indonesia.[16]

In India, the prevalence rate varies in different institutions from 28 to 84%.[17] A study from Coimbatore, Tamil Nadu, showed the presence of ESBLs to be 40% while from Nagpur this figure was 50% in urinary isolates.[18,19] Another comparatively recent study in 2005, from New Delhi, showed 68.78 % of the strains of gram negative bacteria to be ESBL producers.[20]

This study was undertaken in a 500 bedded tertiary care teaching hospital located at Gangtok (capital of Sikkim, India) to find out the prevalence of ESBLs in gram negative bacilli isolated from in patients and their antibiotic susceptibility pattern as well as to see whether routine detection of ESBLs is necessary. Although the CLSI recommends the combined disc method and MIC broth micro-dilution for ESBL detection,[7] in our study, we used DDST as a screening method and cephalosporin/clavulanate combination discs as phenotypic confirmatory test to detect ESBLs. Disc diffusion method is easy to perform and it is comparatively simple and cost effective.

## MATERIALS AND METHODS

### Study design

Central Referral Hospital is a tertiary healthcare teaching hospital. During the study period from June 2007 to June 2008, 1489 specimens were collected from patients with suspected nosocomial infections, according to definitions described by the Centers for Disease Control (CDC).[21] In particular, infections were considered nosocomial if symptoms and signs appeared after 48 hours of hospitalization. Various samples included in the study were urine, pus, sputum, blood and cerebrospinal fluid.

### Bacterial strains and susceptibility testing

One thousand four hundred and eighty-nine specimens collected from patients with suspected nosocomial infections were cultured on blood agar (Hi media, Mumbai, India) and MacConkey agar (Hi media, Mumbai, India) except for urine samples which were plated on Cysteine Lactose Electrolytes Deficient (CLED) agar (Hi media, Mumbai, India). Isolated strains were identified on the basis of colony morphology and biochemical reactions.[22]

The susceptibility of gram negative bacilli to antimicrobial agents was performed on Muller Hinton agar (Hi media, Mumbai, India) by modified Kirby Bauer disc diffusion method following the criteria put forward by the CLSI,[23] with 30μg each of the third generation cephalosporins (3GCs), ceftazidime, cefotaxime and ceftriaxone. The inoculated plates were incubated for 16-18 hours at 37°C. Isolates found resistant or with decreased susceptibility (Intermediate) to any one of the 3GC antibiotics were selected for the presence of ESBLs.[24]

Antibiogram of each isolate was also determined for the following antimicrobials by modified Kirby Bauer disc diffusion method[25] ampicillin (10μg), ampicillin/sulbactam (10/10μg), piperacillin (100μg), piperacillin/tazobactam (100/10μg), tetracycline (30μg), trimethoprim/sulfamethoxazole (1.25/23.75 μg), ciprofloxacin (5μg), gentamicin (10μg), and imipenem(10μg).

### Testing for presence of ESBL

ESBL detection was carried out by two procedures

#### 1. Screening for ESBL producers - Double disc synergy assay

The DDST was performed as a standard disc diffusion assay on Muller Hinton Agar (MHA). Discs containing 30μg aztreonam and 30μg of ceftazidime, ceftriaxone and cefotaxime each were placed 30mm apart (centre to centre) around a disc containing amoxicillin plus clavulanic acid (augmentin 20μg + 10μg). The MHA plate was incubated at 37°C for 24 hrs. Enhancement of inhibition zone of any one of the test antibiotics towards augmentin disc was regarded as presumptive ESBL production and subjected to phenotypic confirmatory test.[26,27] If the screening test was negative it was repeated placing the discs 20mm apart.[28]

#### 2. Phenotypic confirmatory test

Cephalosporin /clavulanate combination discs: This test was performed on Muller Hinton agar by disc diffusion test as recommended by CLSI. A greater than or equal to five mm increase in zone diameter for either ceftazidime (30μg) or cefotaxime (30μg) tested in combination with clavulanate versus its zone diameter when tested alone confirmed an ESBL producing organism.[27]

### Quality control

Every batch of the media prepared was checked for sterility for 24 hours. *E. coli* ATCC 25922 and *P. aeruginosa* ATCC 27853 were used as quality control strains for antimicrobial susceptibility testing. Quality control when performing screening and phenotypic confirmatory tests: Simultaneous testing with a non-ESBL producing organism *E.coli* ATCC 25922 and an ESBL-producing organism *K. pneumoniae* ATCC 700603 was performed.

## RESULTS

A total of 258 bacteria were isolated from 258 patients. These consisted of 152 urinary tract infections, 70 wound infections, 12 blood stream infections, 22 cases of pneumonia and 2 cases of meningitis. Of these 258 bacterial isolates, 238 were gram negative bacilli and the remaining 20 were *Staphylococcus aureus*. These gram-negative isolates were identified as *Escherichia coli* (n=130), *Klebsiella pneumoniae* (n=35), *Pseudomonas aeruginosa* (n= 46), *Proteus mirabilis* (n=7), *Enterobacter cloacae* (n=8), *Morganella morganii* (n=7) and *Citrobacter freundii* (n=8) [Table 1].

**Table 1:** ESBL positive and non-ESBL gram-negative bacilli in clinical samples

Clinical samples		Blood	Sputum	Pus	Urine	CSF	Total
*E. coli*	ESBL	3	0	5	25	1	34
	Non ESBL	2	5	10	74	5	96
*K. pneumoniae*	ESBL	2	13	2	3	0	20
	Non ESBL	5	2	4	3	1	15
*P. aeruginosa*	ESBL	1	0	1	13	0	15
	Non ESBL	2	0	16	13	0	31
*P. mirabilis*	ESBL	0	0	0	3	0	3
	Non ESBL	0	0	0	4	0	4
*E. cloacae*	ESBL	0	0	0	0	0	0
	Non ESBL	0	0	5	0	0	5
*M. morganii*	ESBL	0	0	0	5	0	5
	Non ESBL	0	0	2	0	0	2
*C. freundii*	ESBL	0	0	0	4	0	4
	Non ESBL	0	0	0	4	0	4

ESBL - Extended spectrum beta lactamase

Out of 238 Gram negative bacilli, 102 showed resistance or decreased susceptibility to any one of the three 3GC. These were then tested for ESBL production by Double disc synergy test and phenotypic confirmatory test. Eighty one isolates were positive for ESBL by both the methods. Of these 34 isolates were *E. coli*, 20 isolates were *K. pneumoniae*, 15 isolates were *P. aeruginosa*, 3 isolates were *P. mirabilis*, 5 isolates were *M. morganii* and 4 isolates were *C. freundii* [Table 1].

We observed that ceftazidime was the most effective in detecting ESBL producers among the third generation cephalosporins [Table 2].

**Table 2:** Resistance of ESBL producing gramnegative bacilli to 3GC and aztreonam to screening tests

ESBL positive bacterial strains	Screening test			
	
	Aztreonam (%)	Cefotaxime (%)	Ceftazidime (%)	Ceftriaxone (%)
*E. coli* (n=34)	24 (70.59)	25 (73.53)	28 (82.35)	20 (58.82)
*K. pneumoniae (n=20)*	14 (70)	15 (75)	16 (80)	13 (65)
*P. aeruginosa* (n=15)	11 (73.33)	11 (73.33)	12 (80)	9 (60)
*P. mirabilis* (n=3)	2 (66.67)	2 (66.67)	3 (100)	2 (66.67)
*M. morganii* (n=5)	3 (60)	3 (60)	5 (100)	3 (60)
*C. freundii* (n=4)	2 (50)	2 (50)	3 (75)	2 (50)

ESBL - Extended spectrum beta lactamase

Our study revealed 100% agreement of the two methods - DDST and phenotypic confirmatory test [Table 3] in detection of ESBL producers.

**Table 3:** Number of ESBL producers detected by screening, confirmatory tests

Bacterial strains	ESBL positive by screening test	ESBL positive by confirmatory test
*E. coli* (n=130)	34	34
*K. pneumoniae* (n=35)	20	20
*P. aeruginosa* (n=46)	15	15
*P. mirabilis* (n=7)	3	3
*E. cloacae* (n=5)	-	-
*M. morganii* (n=7)	5	5
*C. freundii* (n=8)	4	4

ESBL - Extended spectrum beta lactamase

A significant proportion of the ESBL producing strains were found to be resistant to antimicrobial agents including ampicillin (100%), ampicillin/sulbactam (81.29%), piperacillin (70.88%), piperacillin/tazobactam (51.89%), trimethoprim/sulfamethoxazole (78.48%), tetracycline (74.68%), ciprofloxacin (51.89%) and gentamicin (54.43%). Imipenem was found to be the most effective antibiotic against ESBL producers (97.53% of isolates were sensitive), while in non-ESBL producing isolates resistance was nil. ESBL producing isolates were resistant to more antimicrobial agents than non-ESBL producing isolates. The highest rate of resistance in ESBL negative isolates was seen against ampicillin (81.29%) which was significantly (p < 0.01) lower than ESBL producing isolates. This was followed by resistance to ampicillin/sulbactam (78.29%). However, in this case, the difference was not significant (p > 0.05) [Table 4]

**Table 4:** Antibiotic susceptibility pattern of ESBL and non-ESBL isolates

Antibiotics	ESBL (n=81) % resistant	Non ESBL (n=157) % resistant	Difference (p)
Ampicillin	100	81.29	<0.01
Ampicillin + Sulbactam	81.29	78.29	>0.05
Piperacillin	70.88	27.09	<0.01
Piperacillin + Tazobactam	51.89	27.09	<0.01
Cotrimoxazole	78.48	38.06	<0.01
Tetracycline	74.68	39.35	<0.01
Ciprofloxacin	51.89	18.70	<0.01
Gentamicin	54.43	34.19	<0.01
Imipenem	2.47	0	>0.05

ESBL - Extended spectrum beta lactamase

Multidrug resistance was seen in 56 (69.14%) ESBL-positive isolates and 34 (21.66%) non- ESBL isolates. This difference was highly significant (p < 0.01).

## DISCUSSION

This study demonstrates the presence of ESBL-mediated resistance in gram-negative bacilli causing infections in various wards and ICU of a tertiary hospital in Sikkim, India. Although a few studies have reported on the prevalence of ESBL producers in Indian hospitals, ESBL producing bacteria may have evolved in several hospitals all over the country. ESBL detection is not commonly carried out in many microbiology units in developing countries, India included. This could be attributed to lack of awareness and lack of resources and facilities to conduct ESBL identification. The high rate of resistance noted among the isolates in the present study, is of serious concern. Eighty one of the 238 (34.03%) gram-negative bacilli were ESBL producing. In this study, ESBL producing isolates were significantly more resistant to ampicillin (p < 0.01), piperacillin (p < 0.01), cotrimoxazole (p < 0.01), tetracycline (p < 0.01), ciprofloxacin (p < 0.01) and gentamicin (p < 0.01) as compared to non-ESBL producing gram-negative isolates. In our study, resistance to 3GCs was found to coexist with resistance to two or more antibiotics like ampicillin, piperacillin, cotrimoxazole, tetracycline, ciprofloxacin, gentamicin as also reported by Subha *et al*.[29] and Duttaroy *et al*.[30] indicating multidrug resistance pattern. Mechanisms of co-resistance are not clear, but one possible mechanism is the co-transmission of ESBL and resistance to other antimicrobials within the same conjugative plasmids.[31]

The prevalence of ESBL producers varies across continents and countries and also within hospitals.[8–16] In India, the prevalence rate varies in different institutions from 28 to 84%.[17] In our study the prevalence of ESBL was 34.03%. *E. coli* (26.15%); *K. pneumoniae* (57.14%); *P. aeruginosa* (32.61%), *P. mirabilis* (42.86%), *M. morgani* (71.43%), *C. freundii* (50%) were found to be ESBL positive by DDST. On detection of ESBL producers, we saw 100% agreement in DDST and phenotypic confirmatory test [Table 3]. Although the specificity of DDST has been well documented[24,32] its sensitivity has been variably reported as 76.5%,[24] 3%,[33] 87%[34] and 79%[28] in various studies. Various factors like precise placement of the disc, correct storage of the clavulanate containing disc and performance of appropriate control tests are critical to the sensitivity of DDST.[24,34,35] DDST can lack sensitivity because of the problems of optimal disc spacing, the inability of the clavulanate to inhibit all ESBLs and the inability of the test to detect ESBLs in strains producing chromosomal cephalosporinases.[35] To overcome the problem of optimal disc spacing, Thomson and Sanders used the recommended disc spacing of 30mm and then repeated at 20mm to see if the former disc spacing was negative.[28]

By routine disc diffusion susceptibility test, 26.15% of ESBL positive *E. coli*, 57.14% of ESBL positive *K. pneumoniae*, 32.60% of ESBL positive *P. aeruginosa*, 42.85% of ESBL positive *P. mirabilis*, 71.42% of ESBL positive *M. morganii*, 50% of ESBL positive *C. freundii* showed a resistance profile to the 3GCs, indicating that 28.53% to 73.85% of the ESBL isolates would have been reported as susceptible. Researchers also reported the resistance profiles of 58%,[36] 48%[29] and 82% to the 3GCs.[28] Thus it is clear that additional specific tests are required for detection of ESBL enzyme.[28,29]

We agree with observations of previous studies considering ceftazidime to be most effective in detecting ESBL producers among the 3GCs;[34] though some other workers reported maximum ESBL detection rate by ceftriaxone followed by cefotaxime and lastly ceftazidime.[37,38]

The strength of our study is that in our study population the screening test is as good as the phenotypic confirmatory test.

The limitation of our study was that we could not use any advanced molecular methods due to lack of infrastructure.

## CONCLUSION

To sum up, the prevalence of ESBL was found to be 34.03% in our hospital which cannot be ignored. Since ESBL producers were detected with equal efficacy by screening test DDST and phenotypic confirmatory test; and the sensitivity of screening test improved with the use of more than one antibiotic, addition of one or two antibiotics would not increase the cost and labor, we recommend DDST to be used routinely as a screening test using multiple antibiotics in all microbiology units.

## References

[1] Paterson DL, Bonomo RA (2005). Extended-spectrum β-lactamases: A clinical update. Clin Microbiol Rev.

[2] Paterson DL, Ko WC, Von Gottberg A, Casellas JM, Mulazimoglu L, Klugman KP (2001). Outcome of cephalosporin treatment for serious infections due to apparently susceptible organisms producing extended-spectrum beta-lactamases: Implications for the clinical microbiology laboratory. J Clin Microbiol.

[3] Siegel JD, Rhinehart E, Jackson M, Chiarello L, Health Infection Control Practices Advisory Committee (2006). Management of multidrug-resistant organisms in healthcare settings.

[4] Bradford PA (2001). Extended-spectrum beta-lactamases in the 21st century: Characterization, epidemiology, and detection of this important resistance threat. Clin Microbiol Rev.

[5] Thomson KS (2001). Controversies about extended-spectrum and AmpC beta-lactamases. Emerg Infect Dis.

[6] Wayne PA (2008). Performance standards for antimicrobial susceptibility testing; 18th informational supplement. Clin Lab Standards Inst.

[7] Stürenburg E, Mack D (2003). Extended-spectrum beta-lactamases: Implications for the clinical microbiology laboratory, therapy, and infection control. J Infect.

[8] Babini GS, Livermore DM (2000). Antimicrobial resistance amongst *Klebsiella* spp. collected from intensive care units in Southern and Western Europe in 1997-1998. J Antimicrob Chemother.

[9] Hanberger H, Garcia-Rodriguez JA, Gobernado M, Goossens H, Nilsson LE, Struelens MJ (1999). Antibiotic susceptibility among aerobic gram-negative bacilli in intensive care units in 5 European countries. French and Portuguese ICU Study Groups. JAMA.

[10] Günseren F, Mamikoğlu L, Oztürk S, Yücesoy M, Biberoğlu K, Yuluğ N (1999). A surveillance study of antimicrobial resistance of gram-negative bacteria isolated from intensive care units in eight hospitals in Turkey. J Antimicrob Chemother.

[11] Moland ES, Black JA, Ourada J, Reisbig MD, Hanson ND, Thomson KS (2002). Occurrence of newer beta-lactamases in Klebsiella pneumoniae isolates from 24 US hospitals. Antimicrob Agents Chemother.

[12] Borer A, Gilad J, Menashe G, Peled N, Riesenberg K, Schlaeffer F (2002). Extended-spectrum beta-lactamase-producing Enterobacteriaceae strains in community-acquired bacteremia in Southern Israel. Med Sci Monit.

[13] El-Karsh T, Tawfik AF, Al-Shammary F, Al-Salah S, Kambal AM, Shibl A (1995). Antimicrobial resistance and prevalence of extended spectrum β-lactamase among clinical isolates of gram-negative bacteria in Riyadh. J Chemother.

[14] AitMhand R, Soukri A, Moustaoui N, Amarouch H, ElMdaghri N, Sirot D (2002). Plasmid-mediated TEM-3 extended-spectrum beta-lactamase production in Salmonella typhimuium in Casablanca. J Antimicrob Chemother.

[15] Yu Y, Zhou W, Chen Y, Ding Y, Ma Y (2002). Epidemiological and antibiotic resistant study on extended-spectrum betalactamase-producing Escherichia coli and *Klebsiella pneumoniae* in Zhejiang Province. Chin Med J (Engl).

[16] Paterson DL, Bonomo RA (2005). Extended-spectrum betalactamases: A clinical update. Clin Microbiol Rev.

[17] Das A, Ray P, Garg R, Kaur B (2001). Extended spectrum beta-lactamase production in Gram negative bacterial isolates from cases of septicemia. Proceedings of the Silver Jubilee Conference.

[18] Babypadmini S, Appalaraju B (2004). Extended spectrum β-lactamases in urinary isolates of *Escherichia coli* and *Klebsiella pneumoniae*-prevalence and susceptibility pattern in a tertiary care hospital. Indian J Med Microbiol.

[19] Tankhiwale SS, Jalgaonkar SV, Ahamad S, Hassani U (2004). Evaluation of extended spectrum beta lactamase in urinary isolates. Indian J Med Res.

[20] Mohanty S, Singhal R, Sood S, Dhawan B, Das BK, Kapil A (2005). Comparative in vitro activity of beta-lactam/beta-lactamase inhibitor combinations against Gram negative bacteria. Indian J Med Res.

[21] Garner JS, Jarvis WR, Emori TG, Horan TC, Hughes JM, Olmsted RN (1996). CDC definitions for nosocomial infection. APIC Infection Control and Applied Epidemiology: Principles and Practice.

[22] Koneman EW, Allen SD, Janda WM, Schreckenberger PC, Winn WC (1997). Color atlas and textbook of diagnostic microbiology.

[23] National Committee For Clinical Laboratory Standards (2004). NCCLS document M100-S15. Performance standards for antimicrobial susceptibility testing.

[24] Hansotia JB, Agarwal V, Pathak AA, Saoji AM (1997). Extended Spectrum-lactamase mediated resistance to third generation cephalosporins in Klebsiella pneumoniae in Nagpur, central India. Indian J Med Res.

[25] Koneman EW, Allen SD, Janda WM, Schreckenberger PC, Winn WC (1992). Colour Atlas and Textbook of diagnostic microbiology.

[26] Jarlier V, Nicolas MH, Fournier G, Philippon A (1988). Extended broad-spectrum beta-lactamases conferring transferable resistance to newer beta-lactam agents in Enterobacteriaceae: Hospital prevalence and susceptibility patterns. Rev Infect Dis.

[27] (2000). Clinical and Laboratory Standards Institute: 2005 guidelines by CLSI/NCCLS - CLSI informational supplement. Approved standard M100-S15 Wayne, PA.

[28] Thomson KS, Sanders CC (1992). Detection of Extended spectrum beta lactamases in the members of the family Enterobacteriaceae: Comparison of the double disc and three -dimensional test. Antimicrob Agents Chemother.

[29] Subha A, Ananthan S (2002). Extended spectrum beta lactamase (ESBL) mediated resistance to third generation cephalosporins among klebsiella pneumoniae in Chennai. Indian J Med Microbiol.

[30] Duttaroy B, Mehta S (2005). Extended spectrum b lactamases (ESBL) in clinical isolates of Klebsiella pneumoniae and Escherichia coli. Indian J Pathol Microbiol.

[31] Martinez-Martinez L, Pascual A, Jacoby GA (1998). Quinolone resistance from a transferable plasmid. Lancet.

[32] Jarlier V, Nicolas MH, Fournier G, Philippon A (1988). Extended broad-spectrum beta-lactamases conferring transferable resistance to newer beta-lactam agents in Enterobacteriaceae: Hospital prevalence and susceptibility patterns. Rev Infect Dis.

[33] Abigail S, Mathai E, Jesudason MV, John TJ (1995). Ceftazidime resistance among Klebsiella pneumoniae in South India. Indian J Med Res.

[34] Cormican MG, Marshall SA, Jones RN (1996). Detection of ESBL producing strains by the Etest ESBL screen. J Clin Microbiol.

[35] Moland ES, Thomson KS (1994). Extended spectrum beta lactamases of Enterobacteriaceae. J Antimicrob Chemother.

[36] Emery CL, Weymouth LA (1997). Detection and clinical significance of Extended spectrum beta lactamases in a tertiary care medical center. J Clin Microbial.

[37] Coudron PE, Moland ES, Sanders CC (1997). Occurrence and detection of ESBL in members of the family Enterobacteriaceae at a veterans medical center. J Clin Microbial.

[38] Datta P, Thakur A, Mishra B, Gupta V (2004). Prevalence of clinical strains resistant to various beta lactamase in a tertiary care hospital in India. Jpn J Infect Dis.

